# Mild SARS-CoV-2 Illness Is Not Associated with Reinfections and Provides Persistent Spike, Nucleocapsid, and Virus-Neutralizing Antibodies

**DOI:** 10.1128/Spectrum.00087-21

**Published:** 2021-09-01

**Authors:** Charles F. Schuler, Carmen Gherasim, Kelly O’Shea, David M. Manthei, Jesse Chen, Cristyn Zettel, Jonathan P. Troost, Andrew A. Kennedy, Andrew W. Tai, Donald A. Giacherio, Riccardo Valdez, James L. Baldwin, James R. Baker

**Affiliations:** a Division of Allergy and Clinical Immunology, Department of Internal Medicine, University of Michigan, Ann Arbor, Michigan, USA; b Mary H. Weiser Food Allergy Center, University of Michigan, Ann Arbor, Michigan, USA; c Department of Pathology, University of Michigan, Ann Arbor, Michigan, USA; d Michigan Nanotechnology Institute for Medicine and Biological Sciences, University of Michigan, Ann Arbor, Michigan, USA; e Michigan Institute for Clinical and Health Research, University of Michigan, Ann Arbor, Michigan, USA; f Division of Gastroenterology, Department of Internal Medicine, University of Michigan, Ann Arbor, Michigan, USA; g Department of Microbiology and Immunology, University of Michigan, Ann Arbor, Michigan, USA; Brigham and Women’s Hospital, and Harvard Medical School

**Keywords:** COVID-19, SARS-CoV-2, antibody, serology, spike, nucleocapsid, immunity, vaccine, persistence, COVID-19, immunoserology, pseudoviral neutralization, viral neutralization

## Abstract

Uncertainty exists whether mild COVID-19 confers immunity to reinfection. Questions also remain regarding the persistence of antibodies against SARS-CoV-2 after mild infection. We prospectively followed at-risk individuals with and without SARS-CoV-2 for reinfection and monitored the spike and nucleocapsid antibodies. This prospective cohort study was conducted over two visits, 3 to 6 months apart, between May 2020 and February 2021. Adults with and without COVID-19, verified by FDA EUA-approved SARS-CoV-2 RT-PCR assays, were screened for spike and nucleocapsid antibody responses using FDA EUA-approved immunoassays and for pseudoviral neutralization activity. The subjects were monitored for symptoms, exposure to COVID-19, COVID-19 testing, seroconversion, reinfection, and vaccination. A total of 653 subjects enrolled; 129 (20%) had a history of COVID-19 verified by RT-PCR at enrollment. Most had mild disease, with only three requiring hospitalization. No initially seropositive subjects experienced a subsequent COVID-19 infection during the follow-up versus 15 infections among initially seronegative subjects (infection rates of 0.00 versus 2.05 per 10,000 days at risk [*P* = 0.0485]). In all, 90% of SARS-CoV-2-positive subjects produced spike and nucleocapsid responses, and all but one of these had persistent antibody levels at follow-up. Pseudoviral neutralization activity was widespread among participants, did not decrease over time, and correlated with clinical antibody assays. Reinfection with SARS-CoV-2 was not observed among individuals with mild clinical COVID-19, while infections continued in a group without known prior infection. Spike and nucleocapsid COVID-19 antibodies were associated with almost all infections and persisted at stable levels for the study duration.

**IMPORTANCE** This article demonstrates that people who have mild COVID-19 illnesses and produce antibodies are protected from reinfection for up to 6 months afterward. The antibodies that people produce in this situation are stable for up to 6 months as well. Clinical antibody assays correlate well with evidence of antibody-related viral neutralization activity.

## INTRODUCTION

Severe acute respiratory syndrome coronavirus-2 (SARS-CoV-2), responsible for coronavirus disease 19 (COVID-19), has caused a pandemic with millions of cases and deaths worldwide (1). While real-time PCR (RT-PCR) and antibody testing for the virus identify acute and resolved SARS-CoV-2 infections (2, 3), questions remain regarding whether infection with this virus provides immunity from reinfection. A large population study examining RT-PCR data suggested that infections continue in a significant portion of individuals following the initial infection, especially in those over 65 years of age (4). However, this study did not look at clinical evidence of reinfection and did not examine antibodies with reinfection. There was also no attempt to correlate reinfection with evidence of immunity to SARS-CoV-2.

Other, smaller studies have examined the presence of antibodies with the likelihood of reinfection and suggested that the presence of some antibody persistence (5) was associated with a reduced risk of reinfection (4, 6, 7). These were not prospective studies, and the antibody levels were not quantitative, making the extent of antibody persistence related to reinfection difficult to evaluate. One study in health care workers demonstrated reduced reinfections among all baseline infected participants but did not stratify by infection severity (8). There have also been questions as to whether nucleocapsid antibodies predict protection to the same degree as spike antibodies (9). Because of these concerns, it remains unclear whether antibody responses after mild infection, commonly defined as an infection not requiring hospitalization (10), are durable and protective (11) and whether a mild RT-PCR-confirmed infection clearly protects an individual from clinical reinfection (4, 6, 7). Given the ongoing worldwide vaccination programs for COVID-19, natural history studies of reinfection and infection-induced, rather than vaccine-induced, antibody effects will become increasingly difficult to perform.

In this prospective cohort study, we evaluated evidence of COVID-19 infection in individuals with and without prior RT-PCR-defined COVID-19 illness and evaluated semiquantitative spike and nucleocapsid antibody titers with pseudoviral neutralization assays in an at-risk population over an extended period of high COVID-19 community transmission.

## RESULTS

We prospectively enrolled 653 subjects. Of them, 129 (20%) had a history of RT-PCR-confirmed SARS-CoV-2, and 209 (32%) had a known negative SARS-CoV-2 RT-PCR, while the remainder (315; 48%) had no history of a clinical illness or positive RT-PCR. The median age was 39 years old, 72% were female, and all were University of Michigan (U-M) health care workers or patients (Table 1). The mean time from a positive RT-PCR to enrollment was 51 days (range, 12 to 120 days). The mean time to follow-up (visit 2) was 126 days. Among those with RT-PCR-confirmed COVID-19, 96% had symptomatic infections (Table 2). The most common symptoms included chills, cough, headache, and myalgia. Among the confirmed COVID-19 cases, only three participants required hospitalization; the rest were treated as outpatients.

**TABLE 1:** Baseline characteristics of participant groups at study entry^*a*^

Characteristic^*b*^		SARS-CoV-2 RT-PCR at baseline		
	Overall	Positive	Negative	No test
*N*	653	129	209	315
Age (yrs)				
Mean (SD)	40.7 (12.1)	42.8 (12.4)	40.1 (12.8)	40.2 (11.4)
Median (IQR)	39 (31, 51)	43 (32, 52)	39 (30, 50)	37 (31, 49)
Sex				
Female	472 (72)	92 (71)	146 (70)	234 (74)
Male	176 (27)	36 (28)	59 (28)	81 (26)
Other	3 (0)	1 (1)	2 (1)	0 (0)
Unknown/not reported	3 (0)	0 (0)	2 (1)	1 (0)
Race				
American Indian/Alaska Native	1 (0)	0 (0)	1 (0)	0 (0)
Asian	57 (9)	10 (8)	11 (5)	36 (11)
Black or African American	26 (4)	10 (8)	6 (3)	10 (3)
Native Hawaiian/other Pacific Islander	2 (0)	0 (0)	1 (0)	1 (0)
White	545 (83)	104 (81)	179 (86)	262 (83)
More than one race	18 (3)	5 (4)	8 (4)	5 (2)
Unknown/not reported	5 (1)	0 (0)	3 (1)	2 (1)
Ethnicity				
Hispanic or Latino	32 (5)	14 (11)	6 (3)	12 (4)
Not Hispanic or Latino	618 (94)	115 (89)	202 (97)	301 (95)
Unknown/not reported	4 (1)	0 (0)	1 (0)	3 (1)
Preexisting medical conditions?				
Yes	139 (21)	39 (30)	51 (24)	49 (16)
No	509 (78)	89 (69)	157 (75)	263 (83)
Unknown/not reported	6 (1)	1 (1)	1 (0)	4 (1)
Chronic lung disease (asthma/emphysema/COPD)	66 (10)	14 (11)	25 (12)	27 (9)
Cardiovascular disease	14 (2)	7 (5)	5 (2)	2 (1)
Diabetes mellitus	22 (3)	10 (8)	5 (2)	7 (2)
Hypertension	66 (10)	20 (16)	21 (10)	25 (8)
Immunocompromised condition	5 (1)	1 (1)	3 (1)	1 (0)
Liver disease	1 (0)	0 (0)	1 (0)	0 (0)
Neurologic/neurodevelopmental/intellectual disability	1 (0)	0 (0)	1 (0)	0 (0)
Chronic renal disease	3 (0)	0 (0)	2 (1)	1 (0)
Other chronic diseases	5 (1)	2 (2)	2 (1)	1 (0)
If female, currently pregnant	0 (0)	0 (0)	0 (0)	0 (0)
Current smoker	17 (3)	3 (2)	9 (4)	5 (2)
Former smoker	98 (15)	27 (21)	38 (18)	33 (10)
BMI				
Mean (SD)	27.7 (8.2)	30.2 (12.0)	27.8 (7.7)	26.6 (6.2)
Median (IQR)	25.7 (22.8, 29.9)	27.3 (23.8, 34.0)	25.8 (22.8, 30.6)	25.1 (22.8, 29.0)

a For all variables, unless otherwise specified, numbers in parentheses are a percentage of the total.

b SD, standard deviation; IQR, interquartile range; COPD, chronic obstructive pulmonary disease.

**TABLE 2:** Symptoms among known COVID-19-positive and -negative participants^*a*^

Characteristic	COVID-19 RT-PCR entry result	
	Positive	Negative
Any symptom present	124 (96)	112 (54)
Abdominal pain	33 (26)	16 (8)
Anosmia (loss of smell)	82 (64)	9 (4)
Chills	92 (71)	45 (22)
Cough (new onset or worsening of chronic cough)	92 (71)	63 (30)
Diarrhea (>3 loose/looser than normal stools per 24-h period)	69 (53)	31 (15)
Dysgeusia (loss of/decrease in taste)	75 (58)	9 (4)
Fever of >100.4°F (38°C)	59 (46)	14 (7)
Headache	96 (74)	65 (31)
Myalgia	95 (74)	47 (22)
Nausea or vomiting	42 (33)	20 (10)
Rhinorrhea	63 (49)	59 (28)
Subjective fever (felt feverish)	77 (60)	29 (14)
Dyspnea	67 (52)	34 (16)
Sore throat	65 (50)	71 (34)

a For all variables, unless otherwise specified, numbers in parentheses are percentage of total.

**FIG 1:**
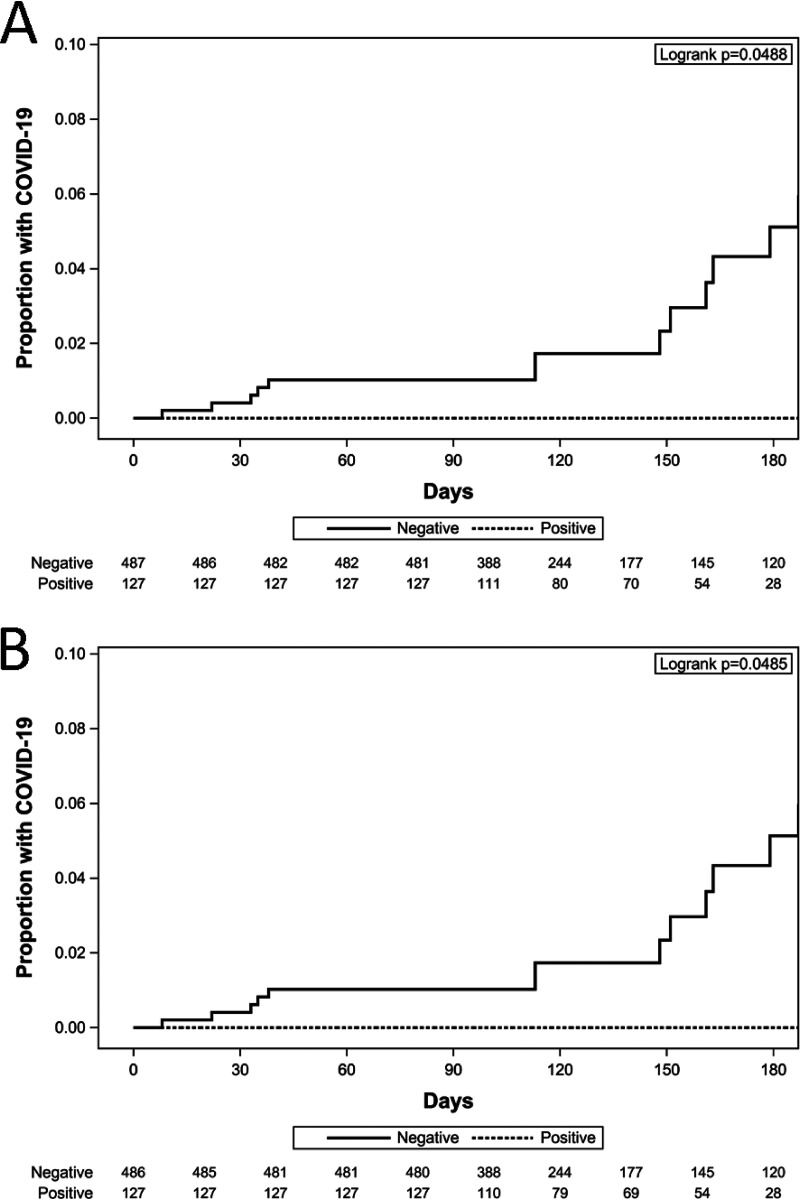
(A) Baseline spike antibody status; (B) baseline nucleocapsid antibody status. Note: While there was high concordance between the spike and nucleocapsid (kappa = 0.93), the 127 positives for spike are not the same 127 positives for nucleocapsid: there were 14 discordant cases (7 + spike/ − nucleocapsid; and 7 − spike/ + nucleocapsid).

Among the subjects with a baseline positive S and/or N antibody result, there were zero SARS-CoV-2 infections during the observation period (Fig. 1A and B; 0 per 10,000 days at risk), compared to antibody-negative subjects, in whom 15 SARS-CoV-2 infections occurred during the same period (Fig. 1A and B; 2.05 per 10,000 days at risk), a significant difference (*P* = 0.0488 for S antibody; *P* = 0.0485 for N antibody). For reinfection analyses, the first date of vaccination was considered to be the end of the observation period, so these results only include data from the time individuals were unvaccinated.

**FIG 2:**
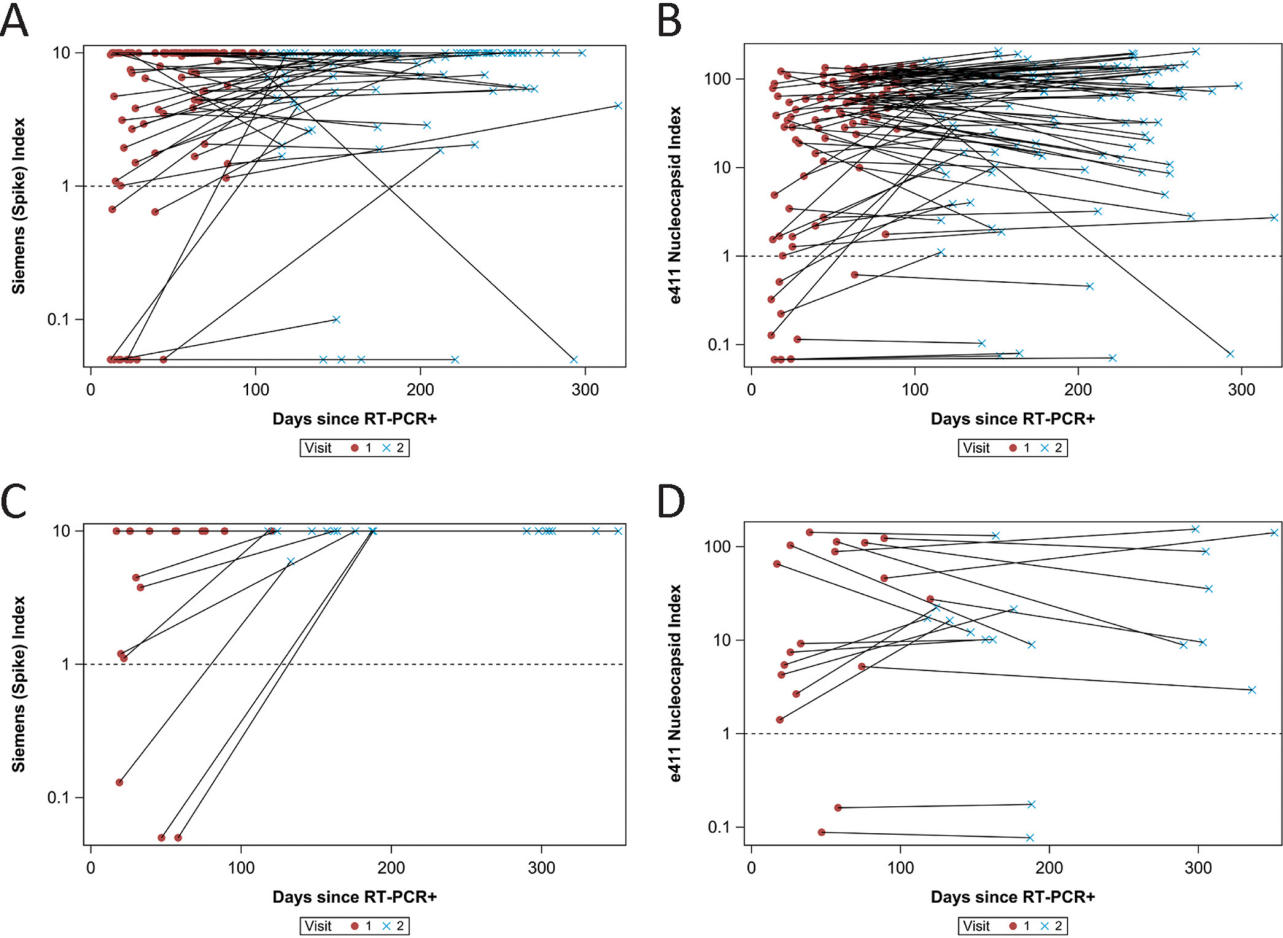
(A) Spike antibody immunoassay index values for RT-PCR-positive, unvaccinated subjects, *n* = 99; (B) nucleocapsid antibody immunoassay index values for RT-PCR-positive, unvaccinated subjects, *n* = 99; (C) spike antibody immunoassay index values for RT-PCR-positive, vaccinated subjects, *n* = 18; (D) nucleocapsid antibody immunoassay index values for RT-PCR-positive, vaccinated subjects, *n* = 18.

Among the RT-PCR-positive subjects, 91% were found to have N antibodies and 90% S antibodies with the higher complexity tests (Fig. 2A and B). Seven subjects produced only detectable N antibodies, and 7 others produced only S antibodies. Among the RT-PCR-negative subjects, 1% had N antibodies and 2% S antibodies with these tests. Among the RT-PCR-positive subjects who produced N and/or S antibodies, the mean levels were unchanged at follow-up (Fig. 2A and B). Furthermore, there was no evidence of an overall decrease in the N or S antibody levels at later times, and only one subject had a significant decrease in N and S antibodies during the study (Fig. 2A and B); vaccinated individuals were not included in this follow-up analysis, as the currently approved vaccines induce an S protein antibody response (12, 13). At the time of visit 2, 169 (26%) of our subjects had been vaccinated. All subjects who were vaccinated received either the BNT162b2 or mRNA-1273 vaccines (12, 13). Not surprisingly, among the RT-PCR-positive subjects who received vaccines, the S antibody but not N antibody levels rose after vaccination (Fig. 2C and D).

**FIG 3:**
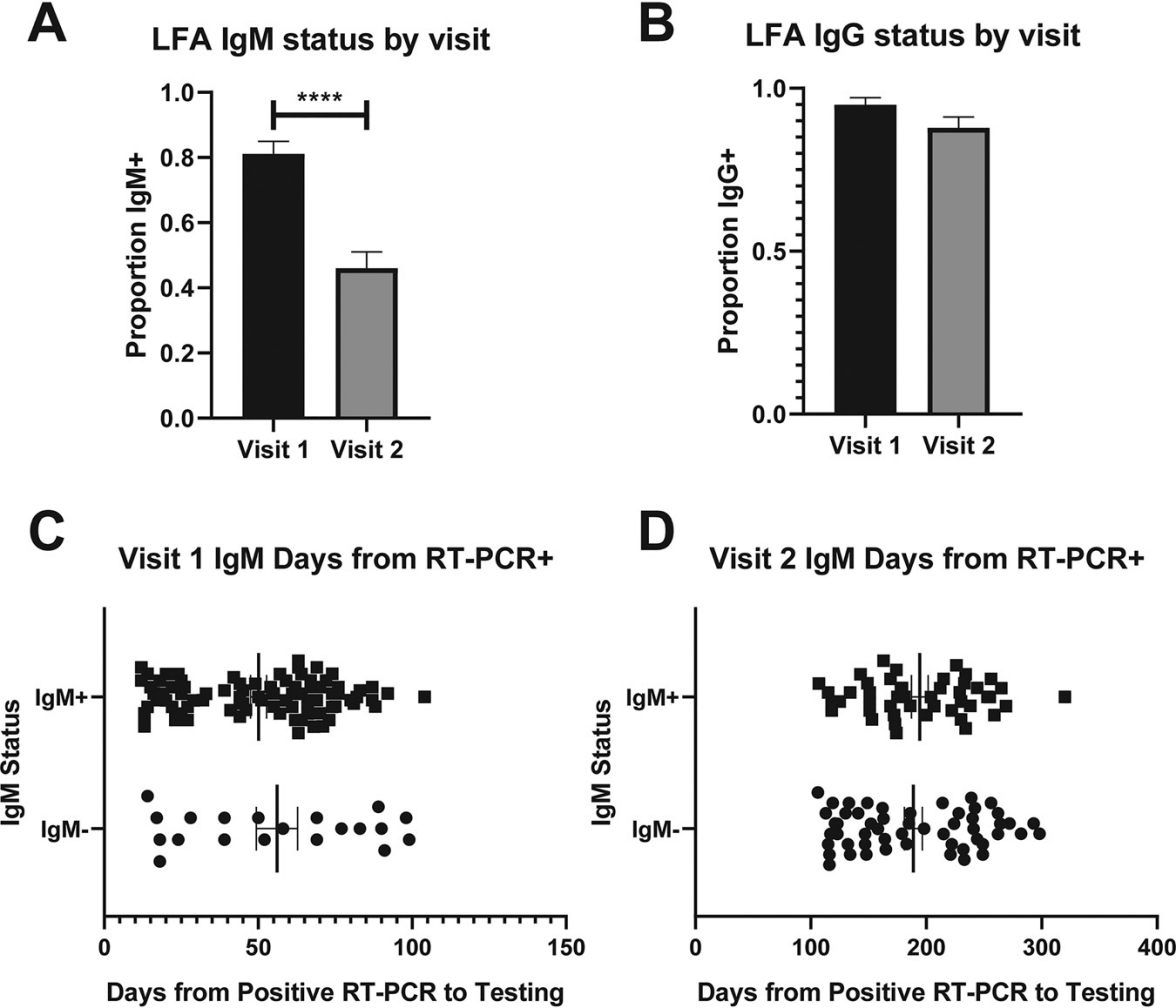
LFA-positive response rate stratified by visit for IgM (A) and IgG (B) among all subjects with a positive RT-PCR for SARS-CoV-2 on study entry. LFA IgM response rate over time for visit 1 (C) and visit 2 (D). ****, *P* < 0.0001.

We evaluated IgM and IgG responses to the SARS-CoV-2 spike protein using the LFA. Among the RT-PCR-positive subjects who did not undergo vaccination during the study, IgM responses decreased significantly from visit 1 to visit 2 (Fig. 3A), while the IgG responses were statistically unchanged (Fig. 3B). At visits 1 and 2, participants with and without IgM responses were not sampled at significantly different time points (Fig. 3C and D), and positive IgM responses were detected over 200 days from the first known infection data using the LFA (Fig. 3D).

**FIG 4:**
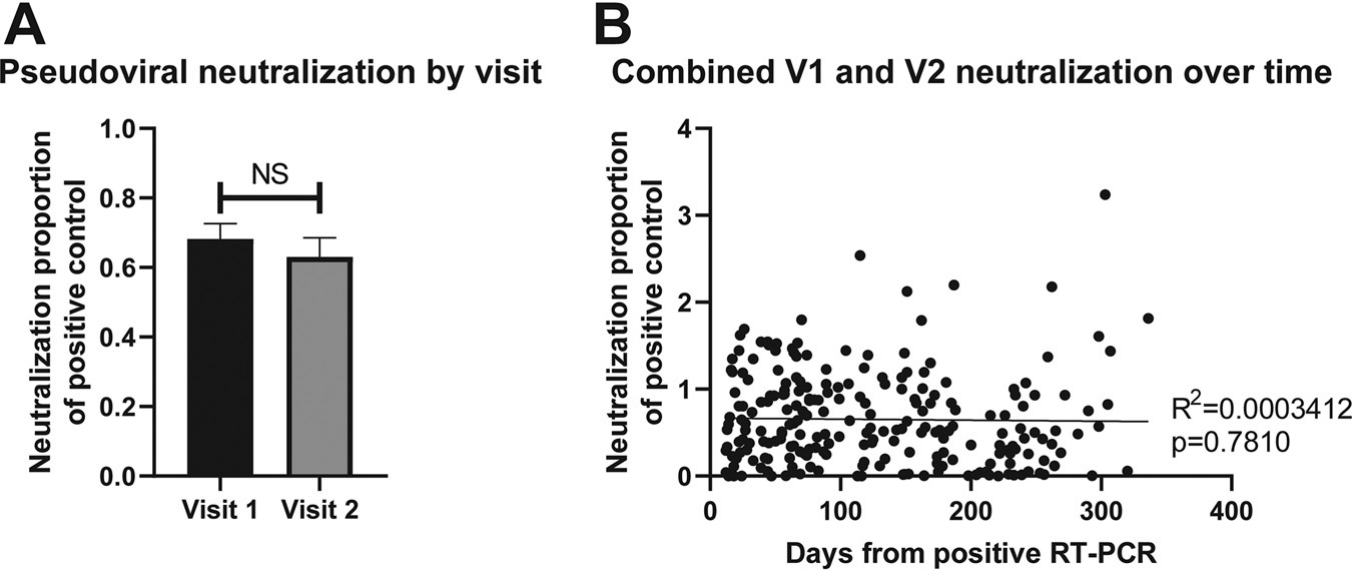
(A) Average pseudoviral neutralization activity as a proportion of the positive control for visit 1 and visit 2. The neutralization proportion of the positive control was calculated by dividing the neutralization activity of each sample over the baseline by the neutralization activity of the positive control over the baseline. (B) Pseudoviral neutralization activity plotted against time from positive RT-PCR for SARS-CoV-2. The trend line represents a simple linear regression and is not significant. NS, not significant.

We also evaluated pseudoviral neutralization activity as a proportion of a positive control for all samples with a baseline positive RT-PCR at entry. The average neutralization activity did not statistically differ from visit 1 to visit 2 (Fig. 4A). In addition, we plotted the neutralization activity over time from positive RT-PCR assays from both visits 1 and 2 combined and noted no significant decrease in the neutralization activity over time (Fig. 4B). The pseudoviral neutralization activity correlated significantly positively at both visits 1 and 2 with both spike and nucleocapsid index values (Fig. S1A-D in the supplemental material).

## DISCUSSION

In this prospective cohort study, no subject with a known SARS-CoV-2 infection had a reinfection during the observation period. In addition, those subjects with who were found to have N or S antibodies to SARS-CoV-2 on lab-based immunoassays but who did not have a known positive RT-PCR at enrollment also had no infections during the observation period. SARS-CoV-2 was spreading in the local community during the course of this study, as evidenced by the infection rates of 25/100,000 reported in Washtenaw County, Michigan, during the study period (14) and signs of infection in the baseline non-RT-PCR-positive group. In addition, all participants had occupational and/or community exposure to SARS-CoV-2. The lower rate of infection in the group without a history of SARS-CoV-2 infection compared to the general population was likely due to the fact that these individuals were practicing strict avoidance measures, including personal protective equipment (PPE) (15–17). The differences in these groups would likely have been greater if not for those precautions. Additionally, most of the infections we observed were mild, as only three subjects with COVID-19 required hospitalization (10). Even with mild infections, however, 90% of subjects with a RT-PCR-confirmed infection produced N and/or S antibodies, and none of these experienced a reinfection. Furthermore, pseudoviral neutralization activity was prominent in this sample set and correlated positively with the clinical spike and nucleocapsid antibody measures.

This study provides strong prospective evidence for longer-term immunity for those infected with SARS-CoV-2 who produce an immune response to mild infection. The present work is consistent with three prior reports (4, 6–8); however, prior work in this area has relied exclusively on retrospective databases (4, 7), utilized only spike protein antibodies (6), used only a history of infection without considering antibodies (4), or not stratified according to infection severity (8). To our knowledge, this is the first report of a prospective study following individuals at risk for COVID-19 to demonstrate a prospective risk reduction for clinical reinfection with a history of mild infection. The protective effect in this study was detected using S and N antibody immunoassays and correlated with pseudoviral neutralization activity, also a distinctive feature of this study. While some studies have suggested that antibodies against SARS-CoV-2 wane over time (9, 18, 19), this study documents the persistence of S or N antibody levels and pseudoviral neutralization activity for up to 6 months after mild SARS-CoV-2 infection. Only one subject in this study lost S and N antibody responses between visits 1 and 2. The second visit occurred nearly 300 days after infection. One concern could be that antibody responses would wane after the time period covered by this study; we are therefore continuing this study with further visits to evaluate whether additional subjects lose antibody responses.

Our study complements the other studies in the literature that have examined the issue of reinfection. Two of these studies looked only at the evidence of virus using nucleic acid amplification tests (4, 7). There was no attempt to determine whether this positive finding was associated with symptoms of clinical illness or with changes in antibody titers. While another recent study did look at recurrent illness in health care workers, its authors performed more of a population-based comparison (6). In addition, this study looked predominantly at spike antibodies as the predictor of clinical and RT-PCR-confirmed reinfection. Another recent study did show reduced reinfections among antibody-positive patients but did not stratify by infection severity (8). We confirm many of the findings of these studies, including the lack of clinical symptoms in individuals with prior antibody detected and protection from reinfections. In addition, while our study is smaller, it is fully prospective and focuses on mild infections only. The individuals were closely monitored in the same location and had identical environments. The COVID-19 RT-PCR-positive group and the group without a history of COVID-19 were closely matched, both geographically and demographically. Therefore, we have a very strong, direct comparison that shows that antibodies to either spike receptor-binding domain or nucleocapsid antigen predicts protection from a subsequent COVID-19 RT-PCR-positive result and especially clinical illness.

In attempting to characterize the immune response in more detail, it should be noted that while the N and S immunoassays cannot differentiate IgG from IgM, the LFA gives separate IgG and IgM data. IgM was detectable by the LFA for an extended period in some participants, up to 300 days after infection in one case. However, IgG was, as expected, the predominant isotype detected by this method, and this supports the notion that anti-S IgG dominates the humoral immune response detected by immunoassay here.

We also evaluated pseudoviral neutralization activity in this sample set. Notably, neutralization activity did not appear to wane over the observation period, as evidenced by Fig. 4B. Furthermore, the pseudoviral neutralization activity correlated positively with commercial antibody measures, consistent with prior reports (20). In particular, the commercial spike receptor-binding domain antibody seemed to correlate best with pseudoviral neutralization (Fig. S1 in the supplemental material). As additional work focuses on viral neutralization measures, connecting such measures with existing commercial assays will become increasingly critical (21). All of these studies, including our own, have limitations. The populations in the National Health Service (NHS) manuscript (6) and our study were young, with an average age of 40 years, and healthy and did not involve high-risk elderly individuals or those with immune problems. There was also little data on the viral variants of SARS-CoV-2 circulating in the areas where these studies were conducted, at the time of these studies. Therefore, we cannot extend these findings beyond the populations we studied or to newer viral variants. In addition, a small group of subjects did produce antibodies despite a history of a negative RT-PCR for SARS-CoV-2; we suspect that these subjects either had false-negative RT-PCR results, false-positive SARS-CoV-2 antibodies, or were sampled outside the window in which virus was detectable. Finally, this study only addresses the humoral response to SARS-CoV-2; subjects without evidence of humoral responses to the virus may indeed be protected through cellular or other immune mechanisms (22–24).

In conclusion, this study shows that subjects with identified N or S antibodies experienced no observed SARS-CoV-2 reinfections over 3 to 6 months’ time. We also demonstrate that N and S antibody responses are observed in 90% of patients with mild SARS-CoV-2 infection. These responses persisted during up to 6 months of follow-up. Together, this indicates that mild SARS-CoV-2 infections lead to seroconversion and protection from recurrent infections. This reinforces vaccination concepts where infected individuals are protected and can delay vaccination for 90 days after infection ends.

## MATERIALS AND METHODS

### Study populations.

This study was approved by the University of Michigan (U-M) Institutional Review Board (HUM00180074), and all subjects gave written informed consent before entering the study. All subjects were either U-M health care workers or patients with a high risk of exposure to COVID-19 and belonged to one of the following three categories:

1. Health care workers providing direct patient care without a history of prior SARS-CoV-2.
2. SARS-CoV-2-positive: Health care workers and patients with a positive reverse transcriptase polymerase chain reaction (RT-PCR) for SARS-CoV-2 from a nasopharyngeal swab on a clinical sample. Subjects could enroll based on a positive RT-PCR run outside U-M if written verification was provided. Enrollment occurred at least 10 days from symptom onset and positive RT-PCR.
3. SARS-CoV-2-negative: Health care workers and patients with a negative COVID-19 RT-PCR within 14 days of enrollment and no prior history of COVID-19.

### Recruitment.

The subjects were recruited via phone or email announcements with a phone follow-up.

### Exclusion criteria.

The exclusion criteria were as follows: immunodeficiency/immunosuppression with known primary or acquired immunodeficiency; anti-rejection therapy following solid organ or bone marrow transplant; biologic therapeutics such as tumor necrosis factor inhibitors; known malignancy and chemotherapy; and systemic immunosuppressive therapy, including corticosteroids equivalent to 20 mg/day of prednisone for 2 weeks.

### Data collection.

Research Electronic Data Capture (REDCap) software (Vanderbilt University) was used for data collection. Subject-reported and electronic medical record (EMR)-verified demographic, medical history, and medication data were collected. For COVID-19-positive subjects, the infection course, travel history, exposure to COVID-19, and, if applicable, hospital course, including medications, laboratory, and imaging, intensive care needs, mechanical ventilation, and complications, were collected. The subjects’ medical records were used to verify key data.

### Specimen collection and handling.

Ten-milliliter standard phlebotomy blood samples were collected into no-additive collection tubes and refrigerated at 4°C for same-day processing. The samples were centrifuged at 2,000 × *g* for 10 min at 4°C. The serum was transferred into 2-ml aliquots in glass screw-thread vials and stored at −20°C until analysis.

### SARS-CoV-2 nucleocapsid electrochemiluminescence immunoassay and spike (S1-RBD) chemiluminescence immunoassay.

Nucleocapsid (N) antibodies were detected via the Elecsys (Roche) SARS-CoV-2 total antibody assay (“N immunoassay”) on a Cobas e411 analyzer and spike (S1-RBD or “S”) antibodies were detected via the ADVIA Centaur (Siemens) SARS-CoV-2 total (COV2T) assay (“S immunoassay”) on an ADVIA Centaur XPT analyzer in the Clinical Laboratory Improvement Amendments (CLIA)-certified U-M Clinical Pathology Laboratory. The assays detect the total SARS-CoV-2 nucleocapsid or spike receptor-binding domain antibodies via a sandwich electrochemiluminescence immunoassay (ECLIA) and chemiluminescence immunoassay (CLIA). A cutoff index (COI) of >1 is used to report a positive result. The assays detect all SARS-CoV-2 N or S antibodies, including IgG and IgM.

### SARS-CoV-2 lateral flow assay.

A COVID-19 antibody lateral flow assay (LFA) from Healgen Scientific (COVID-19 IgG/IgM rapid test cassette, or “Healgen”) was used to evaluate the presence of IgM antibodies. This test detects SARS-CoV-2 S1-receptor-binding domain spike antibodies. The test was run per the EUA Instructions for Use (25) using subject serum. IgM and IgG are separately detected with this LFA. A faint line was read as positive.

### Pseudoviral neutralization assay.

SARS-CoV-2-pseudotyped lentiviral particles (“pseudoparticles”) were produced by transient transfection of HEK-293T cells. The HEK-293T cells were plated 24 h prior to transfection at 5 × 10^6^ cells per 10-cm culture dish. The transfections were performed using FuGENE HD transfection reagent (Promega) with 250 ng of truncated SARS-CoV-2 spike plasmid, 2,500 ng of psPAX2 plasmid (Addgene 12260), and 2,500 ng pGreenFire1 plasmid-encoding copGFP and luciferase reporter proteins (26). The growth medium was replaced with fresh medium at 24 h, and the supernatant was harvested at 60 and 72 h posttransfection, pooled, and filtered through a 0.45-μm syringe filter prior to storage at −80°C.

To perform the neutralization assays, HEK-293T cells stably expressing human ACE2 were seeded at 9,000 cells per well into clear-bottom, opaque, 96-well plates and incubated at 37°C and 5% CO_2_ for 24 h in DMEM/10% FBS/1% Pen-Strep. Serum samples at a 1:10 dilution were plated in triplicate in 96-well plates with 80 μl of complete DMEM. The negative controls were plated in triplicate, and a known neutralizing nanobody (KC3.ep3) (27) was used as a positive control at 20 ng/ml. Twenty microliters of pseudoparticles with Polybrene (40 μg/ml) was added to each well, and the samples were incubated at 37°C for 1 h. Growth medium was aspirated from the cell culture plates and replaced with the antibody-pseudoparticle mixture. The cells were incubated for 72 h prior to luciferase detection using BrightGlo assay reagent (Promega) and a Synergy 2 (BioTek) plate reader according to the manufacturer’s instructions.

### Statistical analysis.

Descriptive statistics were calculated with medians and interquartile ranges and means and standard deviations. The antibody status at baseline was used to predict the time to COVID-19 infection during follow-up using Kaplan-Meier curves and a log-ranked test. Person-time (days) incidence rates were also calculated as the total number of infection cases divided by the sum of person-days, where each individual contributes person-days from the baseline visit to the date of SARS-CoV2 infection or date of follow-up visit. The date of vaccination was also considered the last follow-up date for reinfection analysis. Spaghetti and Sankey plots were used to display changes in antibody levels over time. Linear mixed-effects models for spike, nucleocapsid, and pseudoviral neutralization activity were used to assess significant changes over time. Spearman *r* correlations were calculated between the clinical antibody indices and pseudoviral neutralization activity. Analyses were performed in SAS V9.4 (SAS Institute Inc., Cary, NC, USA) and Prism V8.0 (GraphPad Inc., San Diego, CA, USA).
