# Supplementary material for: Mild SARS-CoV-2 Illness Is Not Associated with Reinfections and Provides Persistent Spike, Nucleocapsid, and Virus-Neutralizing Antibodies
Source: Microbiol Spectr. 2021 Sep 1;9(2):e00087-21. doi: 10.1128/Spectrum.00087-21 (PMC8557889; doi:10.1128/Spectrum.00087-21)
Supplement: SUPPLEMENTAL FILE 1 — Supplemental material. Download SPECTRUM00087-21_Supp_1_seq4.pdf, PDF file, 2.3 MB. [file spectrum00087-21_supp_1_seq4.pdf]

Supplemental Figure 1: A: Visit 1 spike antibody index plotted against pseudoviral neutralization activity; Spearman $r = 0.5066$, $p < 0.0001$. B: Visit 1 nucleocapsid antibody index plotted against pseudoviral neutralization activity; Spearman $r = 0.3877$, $p < 0.0001$. C: Visit 2 spike antibody index plotted against pseudoviral neutralization activity; Spearman $r = 0.5818$, $p < 0.0001$. D: Visit 2 nucleocapsid antibody index plotted against pseudoviral neutralization activity; Spearman $r = 0.3443$, $p = 0.0002$ (approximate).

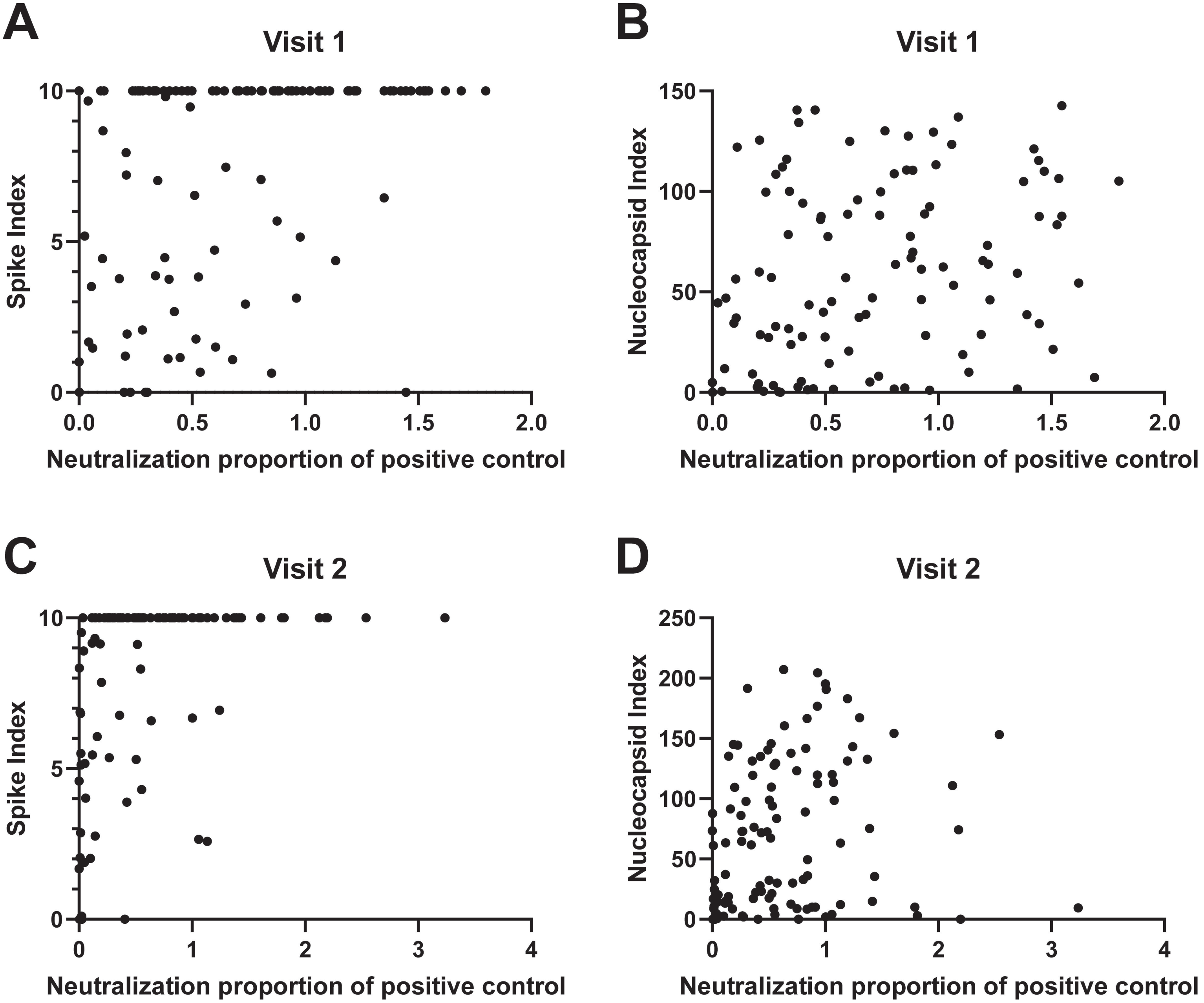
A
Visit 1
Spike Index
Neutralization proportion of positive control
B
Visit 1
Nucleocapsid Index
Neutralization proportion of positive control
C
Visit 2
Spike Index
Neutralization proportion of positive control
D
Visit 2
Nucleocapsid Index
Neutralization proportion of positive control
